# Network meta-analysis of immune-oncology monotherapy as first-line treatment for advanced non-small-cell lung cancer in patients with PD-L1 expression ⩾50%

**DOI:** 10.1177/17588359221105024

**Published:** 2022-06-16

**Authors:** Nick Freemantle, Yingxin Xu, Florence R. Wilson, Patricia Guyot, Chieh-I Chen, Sam Keeping, Gerasimos Konidaris, Keith Chan, Andreas Kuznik, Kokuvi Atsou, Emily Glowienka, Jean-Francois Pouliot, Giuseppe Gullo, Petra Rietschel

**Affiliations:** Comprehensive Clinical Trials Unit, University College London, Institute of Clinical Trials and Methodology, 90 High Holborn 2nd Floor, London WC1V 6LJ, UK; Regeneron Pharmaceuticals, Inc, Tarrytown, NY, USA; Precision HEOR, Vancouver, BC, Canada; Sanofi, Chilly-Mazarin, France; Regeneron Pharmaceuticals, Inc, Tarrytown, NY, USA; Precision HEOR, Vancouver, BC, Canada; Sanofi, Reading, UK; Precision HEOR, Vancouver, BC, Canada; Regeneron Pharmaceuticals, Inc, Tarrytown, NY, USA; Sanofi, Chilly-Mazarin, France; Precision HEOR, Boston, MA, USA; Regeneron Pharmaceuticals, Inc, Tarrytown, NY, USA; Regeneron Pharmaceuticals, Inc, Tarrytown, NY, USA; Regeneron Pharmaceuticals, Inc, Tarrytown, NY, USA

**Keywords:** cemiplimab, cemiplimab monotherapy, IO monotherapy, network meta-analysis, non-small-cell lung cancer, PD-L1 expression, systematic literature review

## Abstract

**Background:**

For patients with advanced non-small-cell lung cancer (NSCLC) and high (⩾50%) programmed cell death-ligand 1 (PD-L1) expression, effective first-line immune-oncology monotherapies with significant survival benefits are approved, cemiplimab being the most recent. In a phase III trial, cemiplimab demonstrated significantly improved overall survival (OS) and progression-free survival (PFS) *versus* chemotherapy in patients with advanced NSCLC and PD-L1 ⩾50%. A systematic literature review and network meta-analysis (NMA) was conducted to identify/compare the efficacy/safety of cemiplimab *versus* pembrolizumab or other immune-oncology monotherapies from randomized-controlled trials (RCTs) published in November 2010–2020.

**Methods:**

Relevant RCTs were identified by searching databases and conference proceedings as per ISPOR, NICE, and Preferred Reporting Items for Systematic Reviews and Meta-Analyses guidelines. NMA with time-varying hazard ratios (HRs) was performed for OS and PFS. Analyses were conducted for objective response rate (ORR) and safety/tolerability. Fixed-effect models were used due to limited evidence. Various sensitivity analyses were conducted to validate the base case analyses.

**Results:**

The feasibility assessment determined that EMPOWER-Lung 1, KEYNOTE-024, and KEYNOTE-042 trials were eligible. IMpower110 was excluded because an incompatible PD-L1 assay (SP142) was used for patient selection. For first-line advanced NSCLC with PD-L1 ⩾50%, cemiplimab was associated with statistically significant improvements in PFS [HR (95% credible interval [CrI]): 0.65 (0.50–0.86), 1–12 months] and ORR [odds ratio (OR) (95% CrI): 1.64 (1.04–2.62)], and comparable OS [HR (95% CrI): 0.77 (0.54–1.10), 1–12 months] *versus* pembrolizumab. There was no evidence of differences between cemiplimab and pembrolizumab for Grade 3–5 adverse events (AEs) [OR (95% CrI): 1.47 (0.83–2.60)], immune-mediated AEs [1.75 (0.33–7.49)], and all-cause discontinuation due to AEs [1.21 (0.58–2.61)].

**Conclusions:**

Considering the limitations of indirect treatment comparisons, in patients with advanced NSCLC and PD-L1 ⩾50%, cemiplimab monotherapy demonstrated significant improvements in PFS and ORR, comparable OS, and no evidence of differences in safety/tolerability *versus* pembrolizumab.

## Introduction

Lung cancer is the second most common cancer in both men and women, and is the leading cause of cancer-related deaths worldwide.^1,2^ Over the last decade, mortality for lung malignancies has exceeded the combined rates of the other most prevalent cancer types including prostate, colon, and breast cancers.^1^ Non-small-cell lung cancer (NSCLC) is the most common type of lung malignancy accounting for 84.3% of all cases in the United States.^3^

In recent years, the treatment paradigm for patients with NSCLC without genomic tumor aberrations [e.g. epidermal growth factor receptor (*EGFR*), anaplastic lymphoma kinase (*ALK*), c-ros oncogene 1 (*ROS1*)]^4^ has evolved in line with an improved understanding of programmed cell death-1 (PD-1) and its ligand (PD-L1) as key regulators of T-cell responses.^5–8^ Patients with advanced NSCLC without such mutations have demonstrated remarkably positive responses to anti-PD-1/PD-L1 treatments.^9–13^ Notably, the approximate prevalence rates of PD-L1 tumor proportion score (TPS) ⩾50% are 18–28%, and for TPS ⩾1%, 47–68%, among patients with advanced NSCLC Stage IIIB or IV, including those with or without *EGFR* mutations and *ALK* alterations.^14–16^

With the approval of various PD-1/PD-L1 immune checkpoint inhibitors for advanced NSCLC, a new class of predictive biomarker assays – complementary and companion diagnostics – has emerged.^17^ Yet, these assays have often differed in many respects across anti-PD-1/PD-L1 trials and treatments (e.g. distinct staining properties and sensitivities).^18^ Such distinctions and potential lack of compatibility across treatments could prevent their interchangeability in clinical use.

A PD-L1 expression threshold of 50% was shown to be optimal using the DAKO 22C3 pharmDx assay for patients using PD-1 inhibitors as monotherapy.^19^ Although the Food and Drug Administration (FDA) indications between the three currently approved, first-line, guideline-recommended PD-1/PD-L1 inhibitors (pembrolizumab, atezolizumab, and cemiplimab) vary in PD-L1 level requirements and assay methods to determine these PD-L1 levels, all are approved for the first-line treatment of advanced NSCLC in patients without certain genomic tumor aberrations.^20–23^ Detailed indications for each treatment are shown in the Supplemental material.

Cemiplimab (cemiplimab-rwlc in the United States) is a highly potent, hinge-stabilized, immunoglobulin G4 100% human monoclonal antibody directed against PD-1. The recent approval of cemiplimab in NSCLC was based on published data from the EMPOWER-Lung 1 trial, in which treatment with cemiplimab resulted in significantly longer overall survival (OS) and progression-free survival (PFS), reducing the risk of death by 43.4% in patients with PD-L1 ⩾50% and by 32.4% in the intention-to-treat (ITT) population *versus* chemotherapy.^24^

While each new treatment has provided significant benefits to patients, the clinical evidence base for trials targeting high PD-L1 expression is still evolving and uncertainty remains regarding the most appropriate first-line therapeutic strategies.^25^ Moreover, there are no trials that directly compare the efficacy and safety between these immunotherapies. Such an analysis might help clinicians to optimize immune-oncology monotherapy especially in patients with high PD-L1 expression.^26^

The objective of this study was to evaluate the comparative efficacy, safety, and tolerability of cemiplimab monotherapy *versus* other immune-oncology monotherapies among patients with locally advanced or metastatic NSCLC with PD-L1 expression ⩾50% who had not received prior systemic therapy for advanced/metastatic disease.

## Patients and methods

### Systematic literature review

Study eligibility criteria are outlined in Table 1. The target population included adult patients with locally advanced or metastatic (Stage IIIB, IIIC, or IV) treatment-naive squamous or non-squamous NSCLC with no known genomic tumor aberrations (e.g. *EGFR*, *ALK*, *ROS1*) and with PD-L1 expression ⩾50%. To capture all relevant clinical studies, the population search strategy was not restricted by PD-L1 expression, PD-L1 scoring assays, or genomic tumor aberrations.

**Table 1.** Population, interventions, comparison, outcomes, and study design.

Criteria	Inclusion criteria	Exclusion criteria
Population^*,$^	• Adult patients (⩾18 years old) with locally advanced or metastatic NSCLC (AJCC Stage IIIB, IIIC, or IV) who were previously untreated with systemic therapy for their locally advanced or metastatic disease (i.e. first line) with PD-L1 ⩾50% Subgroups of interest: • Disease subtype: non-squamous or squamous disease • Smoking status: current or former smoker, or never-smoker • ECOG performance status 0 or 1 • Ethnicity: non-Asian or Asian • Prior treatment experience: newly diagnosed advanced, or progressed from lower stage to advanced stage • Disease stage: metastatic or non-metastatic (locally advanced)	• Pediatric patients (<18 years old) • Patients previously treated with systemic therapy for their locally advanced or metastatic disease (i.e. second or subsequent line)
Interventions^‡^	• Immune checkpoint inhibitor monotherapies ○ Cemiplimab ○ Pembrolizumab ○ Atezolizumab ○ Durvalumab ○ Avelumab • Platinum (carboplatin or cisplatin) in combination with chemotherapy (docetaxel, gemcitabine, paclitaxel, pemetrexed, or vinorelbine), with or without pemetrexed maintenance treatment • Single-agent chemotherapy (docetaxel, gemcitabine, paclitaxel, or vinorelbine) for patients for whom platinum combination therapy was not appropriate • Any systemic interventions other than immune checkpoint inhibitors evaluated in locally advanced NSCLC	• Surgery • Radiotherapy • Neoadjuvant regimens • Adjuvant regimens • Targeted therapy alone or in combination with chemotherapy: *○ EGFR* inhibitors (e.g. gefitinib, erlotinib, afatinib, dacomitinib, icotinib, osimertinib, necitumumab) *○ ALK* inhibitors (e.g. crizotinib, alectinib, ceritinib) *○ BRAF* inhibitors (e.g. dabrafenib, trametinib) ○ *ROS1* inhibitors (e.g. entrectinib, crizotinib)
Comparators	• Any interventions of interest • Placebo or best supportive care • Any treatment that facilitates an indirect comparison	• Interventions not of interest
Outcomes	At least one of the following outcomes: • Efficacy outcomes ○ OS ○ PFS or time to progression ○ Time on treatment ○ Overall response rate ○ Duration of response • Safety outcomes ○ Immune-mediated AEs ○ Grade 3 or 4 AEs (any or specific) ○ Discontinuation due to AEs ○ All-cause mortality ○ HRQoL^§^	• Outcomes not of interest
Study design	• RCTs, phase II or III	• Phase 0, I, or IV trials • Non-RCTs • Observational studies • Single-arm studies • Pooled analyses of RCTs • Case reports, case series • Letters, editorials, press releases, narrative reviews, opinion pieces, etc.
Language	• English-language papers only	• Non-English-language papers (even if abstract is in English)
Time	• Only studies published from 2010 onwards	• Studies published before 2010

* The population used for search strategies and study selection was not restricted further; however, data extraction was not performed for studies in which the entire population consisted of patients with known driver mutations (e.g. *ALK, EGFR, ROS1*).

$ For the purpose of citation screening, any assays used to measure PD-L1 expression were eligible for inclusion.

‡ Studies that exclusively focused on comparisons between different doses, administration regimens, or treatment schedules were excluded.

§ HRQoL data were evaluated and determined not to be feasible for inclusion in the NMA due to variations in how these data were accessed, reported, and analyzed across trials.

AE, adverse event; AJCC, American Joint Committee on Cancer; *ALK*, anaplastic lymphoma kinase; ECOG, Eastern Cooperative Oncology Group; *EGFR*, epidermal growth factor receptor; HRQoL, health-related quality of life; NSCLC, non-small-cell lung cancer; OS, overall survival; PD-L1, programmed cell death-ligand 1; PFS, progression-free survival; RCT, randomized-controlled trial; *ROS1*, c-ros oncogene 1.

Only first-line treatments given as monotherapies that were licensed or those that were in the process of being evaluated by the US FDA at the time of the systematic literature review (SLR) initiation (November 2019) were included. The list of interventions and comparators used for literature screening was based on the National Comprehensive Cancer Network guidelines for NSCLC (version 7.2019),^27^ European Society for Medical Oncology (ESMO) guidelines for NSCLC,^28^ National Institute for Health and Care Excellence (NICE) guidance for advanced NSCLC squamous^29^ and non-squamous,^30^ and the specific treatments listed as comparators in the NICE single technology appraisal of pembrolizumab for untreated PD-L1-positive metastatic NSCLC.^31^

As approval of the first immunohistochemistry (IHC) assay for *in vitro* PD-L1 testing in NSCLC (PD-L1 IHC 22C3 DAKO pharmDx) was granted by the US FDA in 2015,^32,33^ only studies published from 2010 onwards were expected to evaluate patients’ PD-L1 expression level with validated assays and were included in this SLR.

Relevant studies were identified by searching Embase, MEDLINE, and Cochrane Central Register of Controlled Trials with predefined search strategies (see Supplemental material for search strategy). Searches were conducted through the Ovid platform. Database searches were supplemented with searches of specific recent conference proceedings (American Society of Clinical Oncology, ESMO, World Conference on Lung Cancer, European Lung Cancer Conference, and Society for Immunotherapy of Cancer) in addition to a search of the US National Institutes of Health Clinical Trial Registry to identify any ongoing or complete clinical trials that met the inclusion criteria, but that were not yet published. Searches were also supplemented with hand searches of the bibliographies of recent systematic reviews and meta-analyses (i.e. reviews published since 2018), along with a review of any relevant product monographs and drug labels.

Two reviewers, working independently, reviewed all abstracts and proceedings identified by the search according to the selection criteria, except for outcome criteria, which were only applied during the screening of full-text publications. All studies identified as eligible during abstract screening were then screened at a full-text stage by the same two reviewers. Any discrepancies between reviewers were reconciled through discussion, and a third reviewer was included to reach consensus if required. The process of study identification and selection was summarized with a Preferred Reporting Items for Systematic Reviews and Meta-Analyses (PRISMA) flow diagram.^34^

### Network meta-analysis feasibility assessment

A feasibility assessment was conducted to gauge the appropriateness of proceeding with a network meta-analysis (NMA).^35,36^ This process included determination of whether the randomized-controlled trial (RCT) evidence for the interventions formed one connected network, assessment of the distribution of treatments, exploration of the distribution of baseline patient characteristics both within and between comparisons, assessment of outcome definitions and their time points, and exploration of the observed treatment effects to assess variability in outcome reporting. This feasibility assessment process aligned with International Society for Pharmacoeconomics and Outcomes Research, NICE, and PRISMA guidelines.^37–40^

### Statistical analysis

NMAs were performed in a Bayesian framework. Both fixed-effects (FE) and random-effects (RE) models were considered for each analysis.

The posterior distributions of estimated relative treatment effects between the compared interventions obtained with the Bayesian analyses are summarized by the median and 95% credible intervals (CrIs), which were constructed from the 2.5th and 97.5th percentiles of the posterior distributions. CrIs without including 1 in the Bayesian framework are analogous to ‘statistically significant’ by confidence intervals (CIs) in the frequentist framework for hazard ratios (HRs), which is used to describe the NMA findings.

Although the assumptions of an RE model were considered more plausible for the current evidence base, it is not feasible to estimate stable heterogeneity parameters of RE models in which the evidence networks consist of relatively few trials. As a supportive analysis, RE models using informative priors for the between-trial variance were explored according to Turner *et al*.^41^

Comparisons of time-to-event outcomes – OS and PFS – were conducted assuming time-varying HRs *via* fractional polynomial models using data from Kaplan–Meier curves as the base case due to violation of the proportional hazard assumption in several trials. Sensitivity analyses assuming constant HRs were also performed. Comparisons of binary outcomes [e.g. objective response rate (ORR), Grade 3–5 adverse events (AEs)] were performed based on the proportion of patients experiencing the event of interest using a logistic regression model with a binomial likelihood and logit link. All OS data were unadjusted for treatment switching based upon ITT. Safety outcomes were analyzed using the as-treated population from each trial (i.e. patients with any level of PD-L1 expression). Additional details regarding the statistical analysis are provided in the Supplemental material.

Various sensitivity analyses were performed to explore the impact of excluding individual pembrolizumab trials and to investigate the impact of adding atezolizumab to the base case network.

## Results

### SLR

**Figure 1.**
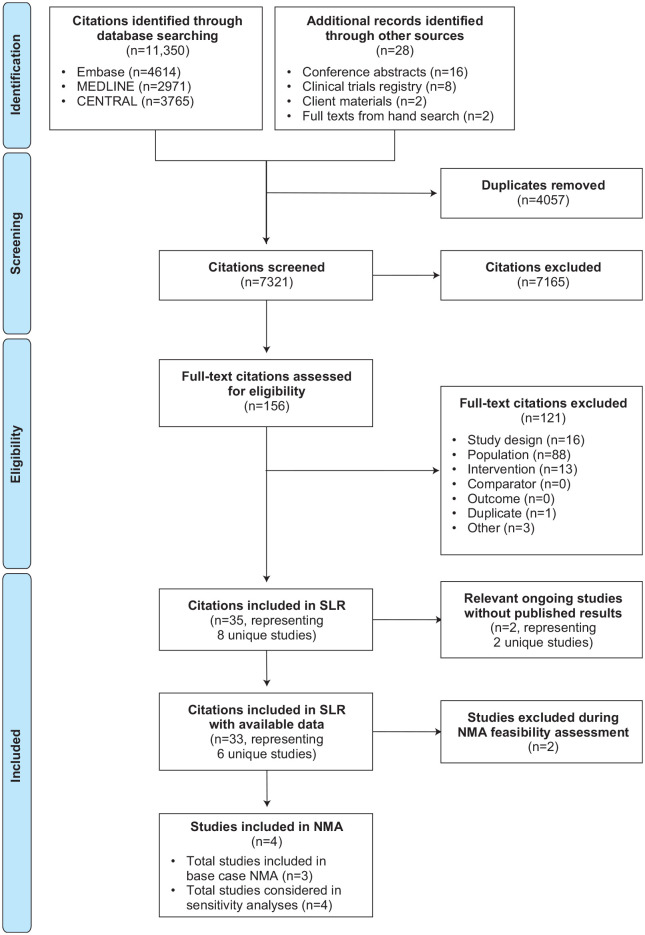
PRISMA flow diagram. NMA, network meta-analysis; PRISMA, Preferred Reporting Items for Systematic Reviews and Meta-Analyses; SLR, systematic literature review.

The SLR was originally conducted in October 2019 and an updated search was performed in November 2020. A total of 35 citations representing eight trials were included from the bibliographic databases and gray literature (e.g. working or white papers, government documents) searches: 9 full-text publications, 16 conference abstracts, 8 registry listings, and 2 sponsor-provided materials. Of these, two registry listings corresponded to two ongoing trials that did not have results available for the population of interest (JAVELIN Lung 100 and PEARL) [ClinicalTrials.gov identifier: NCT02576574; ClinicalTrials.gov identifier: NCT03003962]. This left a total of 33 included citations [ClinicalTrials.gov identifier: NCT02409342; ClinicalTrials.gov identifier: NCT03088540; ClinicalTrials.gov identifier: NCT02142738; ClinicalTrials.gov identifier: NCT02220894; ClinicalTrials.gov identifier: NCT03850444; ClinicalTrials.gov identifier: NCT02453282]^12,24,42–66^ corresponding to six unique trials for final inclusion in the SLR (Figure 1). A follow-up targeted search of the prespecified data sources (i.e. bibliographic databases and conference proceedings) was also performed in May 2021, and no additional trials were identified.

The six trials included in the evidence base were EMPOWER-Lung 1,^24^ KEYNOTE-024,^12^ KEYNOTE-042,^49^ the KEYNOTE-042 China extension study [ClinicalTrials.gov identifier: NCT03850444], IMpower110,^55^ and MYSTIC.^56^ To align with the target population described in Table 1, only data for patients with high PD-L1 expression from the six trials were of interest. Based on the Cochrane Collaboration’s risk of bias assessment, all six trials were considered to have a low risk of bias, except in terms of performance bias, which was high risk given the open-label trial designs.^67^

### NMA and base case

**Figure 2.**
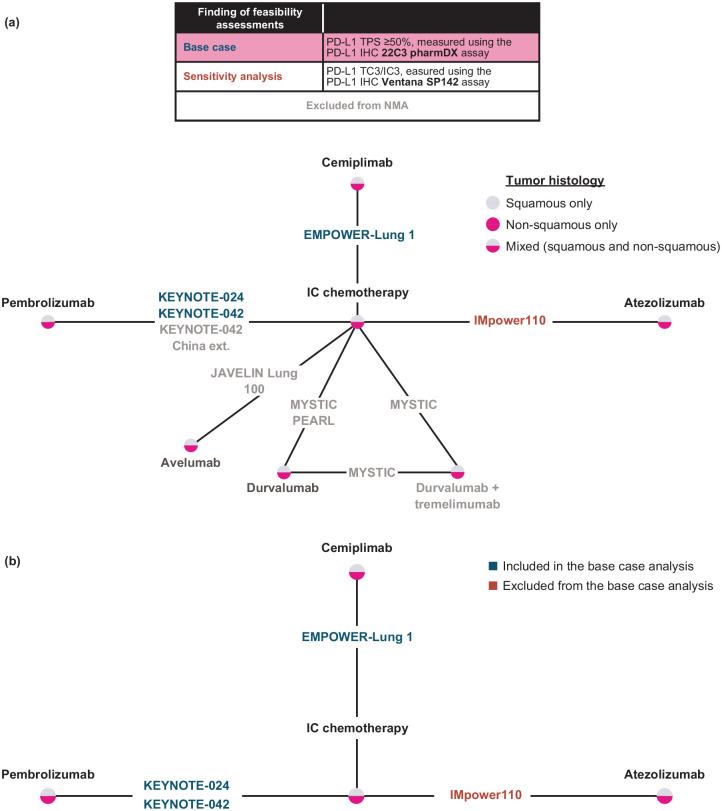
(a) Full evidence network of included and ongoing trials from the SLR and (b) network of base case and sensitivity analysis.* *EMPOWER-Lung 1, KEYNOTE-024, and KEYNOTE-042 were used for the base case while IMpower110 was used for the sensitivity analysis. ext., extension; IC, investigator’s choice; IHC, immunohistochemistry; IO, immune-oncology; NMA, network meta-analysis; NSCLC, non-small-cell lung cancer; PD-L1, programmed cell death-ligand 1; SLR, systematic literature review; TPS, tumor proportion score.

A full evidence network was developed to include all trials that define the relevant evidence base, including the six studies with data available in publications and the two ongoing studies with results not yet available for the population of interest (JAVELIN Lung 100 [ClinicalTrials.gov identifier: NCT02576574] and PEARL [ClinicalTrials.gov identifier: NCT03003962]) [Figure 2(a)]. The evidence network for base case analyses was composed of three trials: EMPOWER-Lung 1, KEYNOTE-024, and KEYNOTE-042 [Figure 2(b)]. For EMPOWER-Lung 1, the PD-L1 ⩾50% population (*n* = 563) instead of the ITT population (*n* = 710) was selected for evaluation of efficacy to align with the indication for cemiplimab monotherapy, while the ITT population was used for safety and tolerability. Similarly, the PD-L1 ⩾50% subgroup (*n* = 599) instead of the ITT population (*n* = 1274) was selected for evaluation of efficacy in KEYNOTE-042 to align with the target population.

The KEYNOTE-042 China extension study was excluded from the analysis due to overlapping patients with KEYNOTE-042. MYSTIC was excluded because durvalumab is not indicated as monotherapy in advanced NSCLC, given MYSTIC failed to meet its primary endpoint.^56^ IMpower110 was only included in the sensitivity analyses because an incompatible PD-L1 assay (SP142) was used for patient selection. Data at the latest follow-up and available in full-text, peer-reviewed publications were preferentially included and used in base case analyses. The extended follow-up data from KEYNOTE-024 at 59.9 months were only available as a conference presentation and were, therefore, included in a sensitivity analysis.

### Baseline patient characteristics

In this evidence base, the distributions of patient age, sex, smoking status, Eastern Cooperative Oncology Group (ECOG) performance status, and prior systemic neoadjuvant or adjuvant therapy were generally similar across trials and were assumed to be comparable; however, some variations were observed for the populations in the base case trials regarding some baseline demographics and clinical characteristics (Table 2).

**Table 2.** Baseline patient characteristics of trials in the base case NMA.

Trial	Treatment	Population or subgroup	*N*	Age, median, years	Male, *n* (%)	Geographic region, *n* (%)		Smoking status, *n* (%)			ECOG performance status, *n* (%)		Histology, *n* (%)		Brain metastases at baseline, *n* (%)
						East Asian	Non-East Asian	Current smoker	Former smoker	Never smoker	0	1	Squamous	Non-squamous	
EMPOWER-Lung 1	Cemi	PD-L1 ⩾50%	283	63.0	248 (87.6)	31 (11.0)	252 (89.0)	105 (37.1)	178 (62.9)	0	77 (27.2)	206 (72.8)	122 (43.1)	161 (56.9)	34 (12.0)
	IC chemo		280	64.0	231 (82.5)	29 (10.4)	250 (89.3)	92 (32.9)	188 (67.1)	0	75 (26.8)	205 (73.2)	121 (43.2)	159 (56.8)	34 (12.1)
KEYNOTE-024	Pembro	ITT, PD-L1 ⩾50%	154	64.5	92 (59.7)	21 (13.6)	133 (86.4)	34 (22.1)	115 (74.7)	5 (3.2)	54 (35.1)	99 (64.3)*	29 (18.8)	125 (81.2)	18 (11.7)
	IC chemo		151	66.0	95 (62.9)	19 (12.6)	132 (87.4)	31 (20.5)	101 (66.9)	19 (12.6)	53 (35.1)	98 (64.9)	27 (17.9)	124 (82.1)	10 (6.6)
KEYNOTE-042	Pembro	Subgroup, PD-L1 ⩾50%^$^	299	63.0	205 (69.0)	92 (31.0)	207 (69.0)	57 (19.0)	178 (60.0)	64 (21.0)	96 (32.0)	203 (68.0)	107 (36.0)	192 (64.0)	19 (6.0)
	IC chemo		300	64.0	210 (70.0)	94 (31.0)	206 (69.0)	59 (20.0)	174 (58.0)	67 (22.0)	91 (30.0)	209 (70.0)	114 (38.0)	186 (62.0)	15 (5.0)

* One patient treated with pembro had an ECOG performance status of 2.

$ Patients with PD-L1 expression ⩾50% were considered the sole primary analysis population prior to 2015 protocol amendment.

cemi, cemiplimab; chemo, chemotherapy; ECOG, Eastern Cooperative Oncology Group; IC, investigator’s choice; ITT, intention-to-treat; mITT, modified ITT; NMA, network meta-analysis; PD-L1, programmed cell death-ligand 1; pembro, pembrolizumab.

In KEYNOTE-024 and KEYNOTE-042, the percentage of male patients ranged from 60% to 70% while in EMPOWER-Lung 1 rates were 82–88%. In KEYNOTE-042, East Asian patients comprised 31% of the population while in KEYNOTE-024 (pembrolizumab arm) and EMPOWER-Lung 1 (cemiplimab arm) rates for this group were 14% and 11%, respectively. Across all three trials (all arms) for the base case, most patients (ranging from 57% to 82%) had non-squamous histology; rates were particularly high in KEYNOTE-024 at 81% and 82% for the pembrolizumab and investigator’s choice (IC) chemotherapy treatment arms, respectively. Lower rates of brain metastases occurred in KEYNOTE-042 (5–6%) and the chemotherapy arm of KEYNOTE-024 (7%) compared with EMPOWER-Lung 1 (12%) and the pembrolizumab arm of KEYNOTE-024 (12%).

The proportion of never-smokers in KEYNOTE-042 was 21% and 22% in the pembrolizumab and chemotherapy arms, respectively. KEYNOTE-024 had lower proportions of never-smokers (3% receiving pembrolizumab and 13% receiving chemotherapy). More former smokers were enrolled in KEYNOTE-024 (67–75%) than in EMPOWER-Lung 1 (63–67%) or KEYNOTE-042 (58–60%). The number of current smokers enrolled was relatively comparable between KEYNOTE-024 (21–22%) and KEYNOTE-042 (19–20%) but was greater in EMPOWER-Lung 1 (33–37%). Prior research using pembrolizumab data shows that the benefit of PD-1 blockage was limited in never-smokers^19,68^; thus, EMPOWER-Lung 1 was limited to current and former smokers.

Although there were some variations in baseline patient characteristics across the three trials included in the base case NMA, results from survival subgroup analyses were generally consistent with HR point estimates favoring immune-oncology monotherapies over IC chemotherapy, with no significant differences (i.e. overlapping CIs) observed across age groups, sex, ECOG performance status, tumor histology, region of enrollment, smoking history or status, or presence of brain metastases. Given the small sample size of each subgroup, lack of time-varying HRs (i.e. Kaplan-Meier curves) on any given subgroup, and given that the distributions of baseline characteristics were generally similar across comparisons and unlikely to be effect modifiers, the NMA was not conducted for subgroups.

### OS

**Figure 3.**
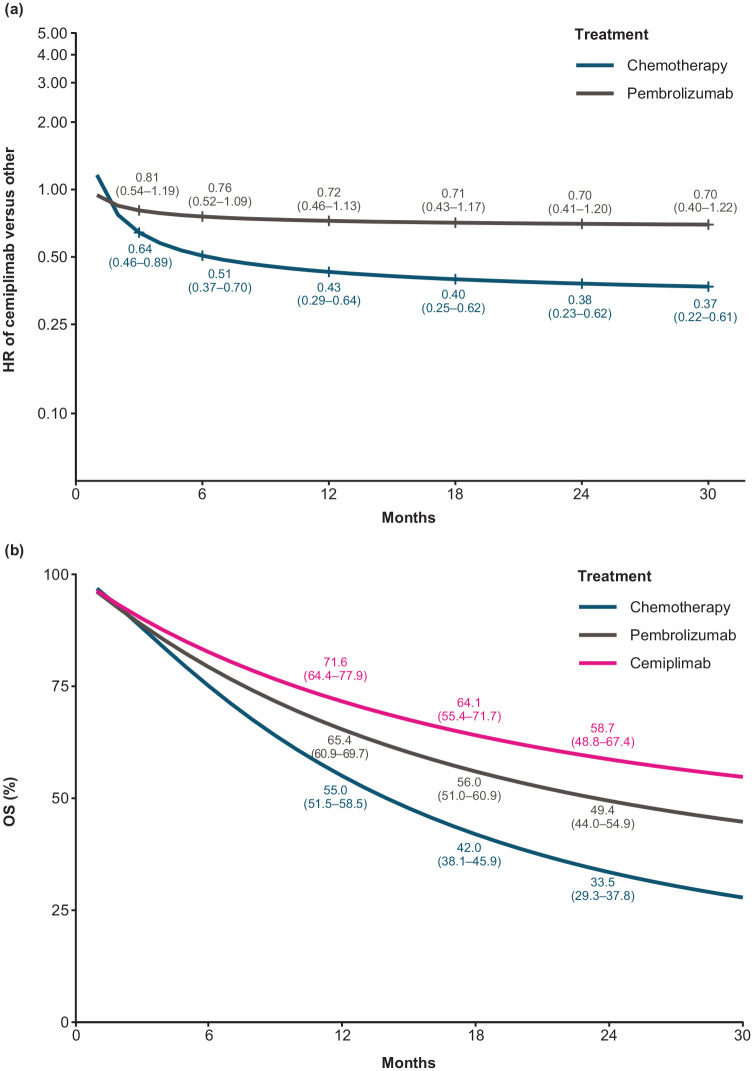
(a) Estimated OS time-varying HRs for cemiplimab *versus* pembrolizumab and chemotherapy, and (b) estimated OS curves for cemiplimab, chemotherapy, and pembrolizumab. Numbers in figures are estimates (95% CrIs). An FE fractional polynomial model NMA was performed as the base case analysis to assess OS for cemiplimab monotherapy *versus* competing interventions. According to the model selection process, the best-fitting model for the base case OS analysis was the FE second-order fractional polynomial with p1 = 1 and p2 = −0.5 (scale and second shape). The estimated time-varying HRs were applied to a pooled reference modeled survival function (IC chemotherapy) to generate the OS proportions over time. CrI, credible interval; FE, fixed effect; HR, hazard ratio; IC, investigator’s choice; NMA, network meta-analysis; OS, overall survival.

The summary of outcomes is provided in Table 3. Among patients with PD-L1 ⩾50%, NMA results showed an improvement in OS with cemiplimab *versus* IC chemotherapy and comparable OS benefit *versus* pembrolizumab. Cemiplimab was consistently associated with statistically significant improvements in OS *versus* IC chemotherapy. OS HRs and 95% CrIs were < 1 for all time points, with the OS benefit increasing steadily from month 3 (HR 0.64, 95% CrI 0.46–0.89) to month 30 (HR 0.37, 95% CrI 0.22–0.61) [Figure 3(a)]. Cemiplimab had comparable OS over time *versus* pembrolizumab (at 3 months: HR 0.81, 95% CrI 0.54–1.19; at 30 months: HR 0.70, 95% CrI 0.40–1.22) [Figure 3(a)]. The estimated time-varying HRs were applied to a pooled reference-modeled survival function (IC chemotherapy) to generate the OS proportions over time [Figure 3(b)]. At 2 years, numerically more patients receiving cemiplimab were alive *versus* those receiving pembrolizumab, and significantly more were alive without progression.

**Table 3.** Summary of outcomes for base case trials: EMPOWER-Lung 1, KEYNOTE-024, and KEYNOTE-042.

Trial	Treatment	Analysis population or subgroup	*N*	Follow-up, median, months	OS, median (95% CI)	OS, HR (95% CI)	PFS, median (95% CI)	PFS, HR (95% CI)	ORR, *n* (%)	Safety population			
										Any level of PD-L1 expression, *n* (%)			
										*n*	Grade 3–5 AEs	Grade 3–5 IMAEs	DAEs
EMPOWER-Lung 1	Cemi	PD-L1 ⩾50%	283	OS, PFS, ORR: 10.8 Safety: 13.1	NR (17.9–NE)	0.57(0.42–0.77)	8.2 (6.1–8.8)	0.54(0.43–0.68)	111 (39.2)	355	132 (37.2)	13 (3.7)	23 (6.5)
	IC chemo		280		14.2(11.2–17.5)	Ref.	5.7 (4.5–6.2)	Ref.	57 (20.4)	342	166 (48.5)	1 (0.3)	14 (4.1)
KEYNOTE-024*	Pembro	ITT, PD-L1 ⩾50%	154	OS, ORR: 25.2 PFS, safety: 11.2	30.0 (18.3–NR)	0.63 (0.47–0.86)	10.3 (6.7–NR)	0.50 (0.37–0.68)	70 (45.5)	154	82 (53.2)	15 (9.7)	17 (11.0)
	IC chemo		151		14.2 (9.8–19.0)	Ref.	6.0 (4.3–6.2)	Ref.	45 (29.8)	150	109 (72.7)	1 (0.7)	16 (10.7)
KEYNOTE-042*	Pembro	Subgroup, PD-L1 ⩾50%	299	OS, PFS, safety: 12.8 ORR: 14.0	20.0 (15.4–24.9)	0.69 (0.56–0.85)	7.1 (5.9–9.0)	0.81 (0.67–0.99)	117 (39.1)	636	–	51 (8.0)	127 (20.0)
	IC chemo		300		12.2 (10.4–14.2)	Ref.	6.4 (6.1–6.9)	Ref.	96 (32.0)	615	–	9 (1.5)	92 (15.0)

* The most mature survival data from peer-reviewed full-text publications were included in base case analyses. Analyses of safety outcomes were performed using data from the follow-up duration comparable to EMPOWER-Lung 1 (i.e. median follow-up 13.1 months). Data reported at the latest time point were considered in the sensitivity analyses.

AE, adverse event; cemi, cemiplimab; chemo, chemotherapy; CI, confidence interval; DAE, discontinuation due to all-cause AE; HR, hazard ratio; IC, investigator’s choice; IMAE, immune-mediated AE; ITT, intention-to-treat; NE, not evaluable; NR, not reached; ORR, objective response rate; OS, overall survival; PD-L1, programmed cell death-ligand 1; pembro, pembrolizumab; PFS, progression-free survival; ref., reference treatment.

Results from the analyses using constant HRs were consistent with the above results and showed that cemiplimab had significantly improved OS *versus* IC chemotherapy (HR 0.57, 95% CrI 0.42–0.77) and comparable OS *versus* pembrolizumab (HR 0.85, 95% CrI 0.60–1.20). Point estimates from the corresponding RE model were consistent with the FE model, although the CrIs were wider.

Among three trials included in the base case analyses, KEYNOTE-024 was the only trial that reported Kaplan–Meier curves for OS at more than one follow-up (median 11.2, 19.1, 25.2, 44.4, and 59.9 months). The result at 25.2 months (defined as final analysis in a peer-reviewed article) was used in the base case NMA with data from the longest follow-up of 59.9 months (presented at a conference) included in the sensitivity analyses. Consistent findings were observed between the base case and sensitivity analyses.

### PFS

**Figure 4.**
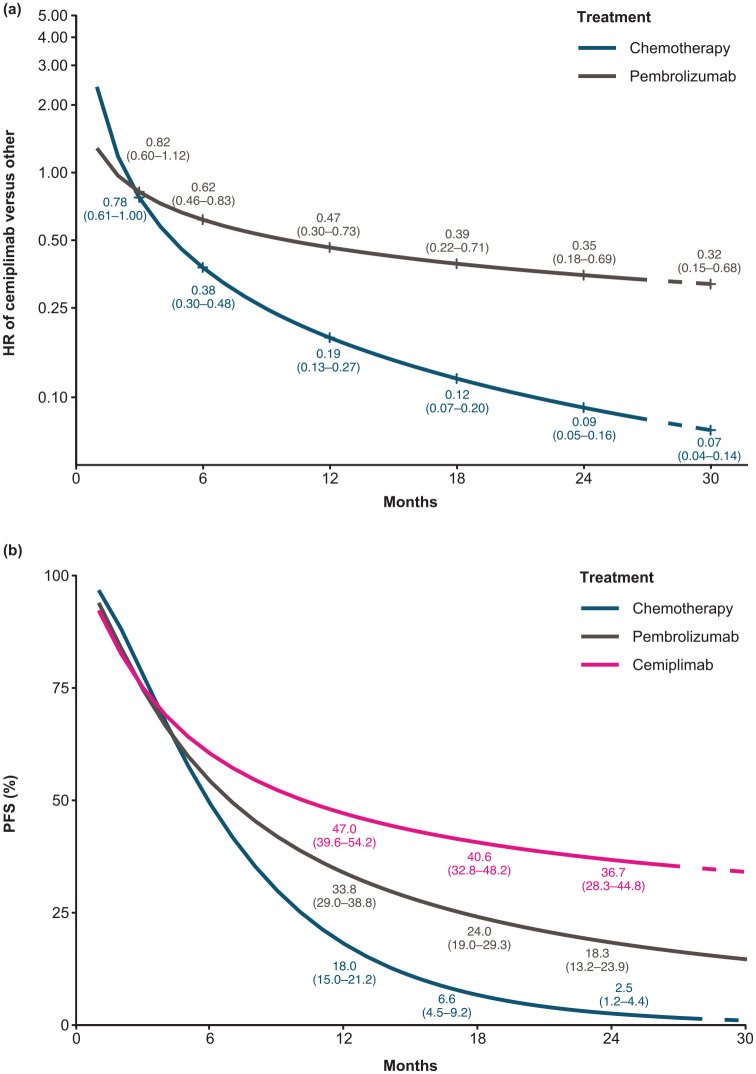
(a) Estimated PFS time-varying HRs for cemiplimab *versus* pembrolizumab and chemotherapy, and (b) estimated PFS curves for cemiplimab, chemotherapy, and pembrolizumab. Numbers in figures are estimates (95% CrI); dashed lines indicate estimates based on model extrapolations. An FE fractional polynomial model NMA was performed as the base case analysis to assess PFS for cemiplimab monotherapy *versus* competing interventions. According to the model selection process, the best-fitting model for the base case PFS analysis was the FE second-order fractional polynomial with p1 = 0 and p2 = −1 (scale and first shape). The estimated time-varying HRs were applied to a pooled reference modeled survival function (IC chemotherapy) to generate the PFS proportions over time. CrI, credible interval; FE, fixed effect; HR, hazard ratio; IC, investigator’s choice; NMA, network meta-analysis; PFS, progression-free survival.

Among patients with PD-L1 ⩾50%, cemiplimab was consistently associated with statistically significant improvements in PFS compared with IC chemotherapy; all PFS HRs and 95% CrIs were <1 for all time points with the PFS benefit increasing from month 3 (HR 0.78, 95% CrI 0.61–0.995) through month 30 (HR 0.07, 95% CrI 0.04–0.14) [Figure 4(a)]. Cemiplimab had statistically significant improvements in PFS *versus* pembrolizumab from month 6 (HR 0.62, 95% CrI 0.46–0.83) through month 30 (HR 0.32, 95% CrI 0.15–0.68) [Figure 4(a)]. The estimated time-varying HRs were applied to a pooled reference-modeled survival function (IC chemotherapy) to generate the PFS proportions over time [Figure 4(b)].

Results from the FE analyses using constant HRs were consistent with the above results and showed that cemiplimab had significantly better PFS *versus* IC chemotherapy (HR 0.54, 95% CrI 0.43–0.68) and comparable PFS *versus* pembrolizumab (HR 0.77, 95% CrI 0.58–1.01). Point estimates from the corresponding RE model were consistent with the FE model, although the CrIs were wider.

Similar to OS data, KEYNOTE-024 reported PFS results at more than one follow-up (median 11.2 and 59.9 months). The result at 11.2 months (from a peer-reviewed article) was used in the base case NMA with data from the longest follow-up of 59.9 months (presented at a conference) included in the sensitivity analyses. Consistent findings were observed between the base case and sensitivity analyses.

### ORRs

Cemiplimab was associated with a statistically significant higher odds of achieving objective response than IC chemotherapy [odds ratio (OR) 2.54, 95% CrI 1.75–3.74)] and pembrolizumab (OR 1.64, 95% CrI 1.04–2.62) (Table 4). Consistent results were seen in corresponding RE models.

**Table 4.** Estimated ORs for ORR, safety, and tolerability for cemiplimab *versus* chemotherapy and pembrolizumab.

ORR.		
IC chemotherapy		
** 1.55 (1.18–2.03)**	Pembrolizumab	
** 2.54 (1.75–3.74)**	**1.64 (1.04–2.62)**	Cemiplimab
Grade 3–5 AEs.		
IC chemotherapy		
** 0.43 (0.26–0.68)**	Pembrolizumab	
** 0.63 (0.46–0.85)**	1.47 (0.83–2.60)	Cemiplimab
Grade 3–5 IMAEs.		
IC chemotherapy		
** 7.07 (3.75–15.15)**	Pembrolizumab	
** 12.58 (2.77–44.34)**	1.75 (0.33–7.49)	Cemiplimab
DAEs.		
IC chemotherapy		
** 1.36 (1.04–1.79)**	Pembrolizumab	
1.63 (0.84–3.35)	1.21 (0.58–2.61)	Cemiplimab

ORs of safety endpoints estimated from FE NMA for patients with any level of PD-L1 expression. Each cell represents the comparison (OR and 95% CrI) of the row treatment *versus* the column treatment. All bolded values are statistically significant at the 0.05 significance level.

AE, adverse event; CrI, credible interval; DAE, discontinuation due to all-cause AE; FE, fixed effect; IC, investigator’s choice; IMAE, immune-mediated AE; NMA, network meta-analysis; OR, odds ratio; ORR, objective response rate; PD-L1, programmed cell death-ligand 1.

### Safety and tolerability

Except KEYNOTE-024, safety data were generally reported at median follow-up of approximately 1 year. The event rates increased slightly for KEYNOTE-024 from the interim analyses at 11.2 months to the latest follow-up at 59.9 months. The base case analyses were performed using data from the most common follow-up duration (i.e. ~12 months).

For Grade 3–5 all-cause AEs, cemiplimab was associated with a lower incidence compared with IC chemotherapy (OR 0.63, 95% CrI 0.46–0.85), with no evidence of a difference, *versus* pembrolizumab (OR 1.47, 95% CrI 0.83–2.60) (Table 4).

For Grade 3–5 immune-mediated AEs (IMAEs), cemiplimab was associated with a greater incidence compared with IC chemotherapy (OR 12.58, 95% CrI 2.77–44.34), with no evidence of a difference, *versus* pembrolizumab (OR 1.75, 95% CrI 0.33–7.49) (Table 4).

For all-cause discontinuations due to AEs (DAEs), cemiplimab was associated with a greater incidence compared with IC chemotherapy (OR 1.63, 95% CrI 0.84–3.35) and pembrolizumab (OR 1.21, 95% CrI 0.58–2.61); however, 95% CrIs included 1, which indicates no statistically significant difference and considerable uncertainty (Table 4).

### Sensitivity analyses: EMPOWER-Lung 1 *versus* KEYNOTE-024 only or KEYNOTE-042 only

Cemiplimab demonstrated statistically significant improvements in OS *versus* IC chemotherapy but showed comparable improvements in OS *versus* pembrolizumab in both KEYNOTE-024-only and KEYNOTE-042-only scenarios. For PFS, cemiplimab was associated with a statistically significant improvement *versus* pembrolizumab and IC chemotherapy in the KEYNOTE-042-only scenario, while cemiplimab was comparable to pembrolizumab in the KEYNOTE-024-only scenario (Table 5). Other sensitivity analyses are included in the Supplemental material.

**Table 5.** Sensitivity analysis: time-varying OS and PFS.

Cemiplimab *versus*	EMPOWER-Lung 1 *versus* KEYNOTE-024 only (excluding KEYNOTE-042)			
	OS, HR (95% CrI)*			
	6 months	12 months	18 months	24 months
IC chemotherapy	**0.51 (0.36–0.70)**	**0.43 (0.28–0.65)**	**0.40 (0.25–0.63)**	**0.38 (0.23–0.62)**
Pembrolizumab	0.81 (0.51–1.26)	0.66 (0.38–1.12)	0.60 (0.32–1.10)	0.57 (0.29–1.08)
	EMPOWER-Lung 1 *versus* KEYNOTE-024 only (excluding KEYNOTE-042)			
	PFS, HR (95% CrI)^$^			
IC chemotherapy	**0.38 (0.30–0.48)**	**0.19 (0.13–0.27)**	**0.12 (0.07–0.20)**	**0.09 (0.05–0.16)**
Pembrolizumab	1.15 (0.75–1.79)	1.04 (0.52–2.14)	0.98 (0.40–2.46)	0.94 (0.33–2.71)
	EMPOWER-Lung 1 *versus* KEYNOTE-042 only (excluding KEYNOTE-024)			
	OS, HR (95% CrI)*			
IC chemotherapy	**0.51 (0.36–0.70)**	**0.43 (0.28–0.64)**	**0.40 (0.25–0.62)**	**0.38 (0.23–0.62)**
Pembrolizumab	0.73 (0.49–1.06)	0.75 (0.47–1.20)	0.77 (0.45–1.30)	0.78 (0.43–1.38)
	EMPOWER-Lung 1 *versus* KEYNOTE-042 only (excluding KEYNOTE-024)			
	PFS, HR (95% CrI)^‡^			
IC chemotherapy	**0.38 (0.30–0.49)**	**0.19 (0.13–0.28)**	**0.13 (0.08–0.21)**	**0.09 (0.05–0.17)**
Pembrolizumab	**0.50 (0.37–0.69)**	**0.39 (0.24–0.63)**	**0.33 (0.18–0.62)**	**0.30 (0.15–0.61)**

Values indicate estimated HRs over time from FE fractional polynomial model NMA. Cells shaded in gray indicate estimates based on model extrapolations. All bolded values are statistically significant at the 0.05 significance level.

* p1 = 1, p2 = −0.5; scale and second shape.

$ p1 = 0, p2 = −1; scale and first shape.

‡ p1 = 0, p2 = −0.5, scale and first shape.

CrI, credible interval; FE, fixed effect; HR, hazard ratio; IC, investigator’s choice; NMA, network meta-analysis; OS, overall survival; PFS, progression-free survival.

## Discussion

In the base case analysis (i.e. assuming time-varying HR *via* fractional polynomial model NMAs) of first-line treatments in patients with locally advanced or metastatic NSCLC with PD-L1 ⩾50%, cemiplimab demonstrated comparable OS and statistically significant improvements in PFS from 6 to 30 months *versus* pembrolizumab, with no evidence of difference in Grade 3–5 all-cause AEs, IMAEs, and all-cause DAEs. The OS and PFS sensitivity analyses assuming constant HRs generally led to similar results.

Results from the sensitivity analyses of OS – excluding the individual pembrolizumab trials – were generally consistent with the base case analysis that included both KEYNOTE-024 and KEYNOTE-042 (i.e. cemiplimab was associated with a comparable OS benefit *versus* pembrolizumab). For PFS, cemiplimab showed a statistically significant lower HR than pembrolizumab in the base case analysis and in the sensitivity analysis with KEYNOTE-042 only, but there was a comparable PFS benefit between cemiplimab and pembrolizumab with KEYNOTE-024 only; this difference appears to be driven by the more favorable PFS result reported for pembrolizumab in KEYNOTE-024 than KEYNOTE-042. When a plausible prior for between-study variation was included, the width of the credibility limits was slightly wider than the FE models, but with similar qualitative inference. Attempts to estimate RE from the available data (e.g. using an uninformative prior) provided very wide credibility limits reflecting the inevitable uncertainty with a sparse network.

Comparisons of safety outcomes in the as-treated populations with any level of PD-L1 expression showed that cemiplimab had a statistically significant lower incidence of Grade 3–5 all-cause AEs *versus* IC chemotherapy while there was no evidence of differences between cemiplimab and pembrolizumab for Grade 3–5 all-cause AEs and Grade 3–5 IMAEs. As expected, cemiplimab, as an immune-oncology agent, had a statistically significant greater incidence of Grade 3–5 IMAEs *versus* IC chemotherapy, while results were comparable with pembrolizumab. For all-cause DAEs, there was no statistically significant difference for cemiplimab compared with IC chemotherapy and pembrolizumab.

In patients with PD-L1 ⩾50%, the OS and PFS results from the clinical trials were less robust for KEYNOTE-042 than KEYNOTE-024. While reasons for this discrepancy remain speculative and have been described extensively by Mok *et al*.,^49^ several potential factors might have contributed to these differences. KEYNOTE-024 was primarily conducted in Europe while the KEYNOTE-042 population was more heterogeneous, predominantly conducted in Asia-Pacific, Eastern Europe, and South America. A disproportionate number of patients may have experienced barriers in access to care, including subsequent therapy, resulting in lower PFS and survival rates independent of the observed treatment effect.^49^ Second, some heterogeneity existed in patient populations between KEYNOTE-042 and KEYNOTE-024 with respect to smoking history and histology. Third, data on tumor mutation burden, including oncogenic drivers other than *EGFR*/*ALK*, were not reported. If substantial differences existed in tumor mutation burden between trial populations, these differences might have confounded the effect of pembrolizumab on survival for either or both studies. Finally, it has been suggested that patients with PD-L1 levels ⩾50% do not comprise a homogeneous patient population, and the balance across trials and trial arms with respect to higher cutoff levels (e.g. 80–90%) remains unknown.^69^

Three trials identified from the SLR were excluded from the base case NMA for various reasons. Given that the KEYNOTE-042 China extension study was exclusively conducted in China and the population overlapped with KEYNOTE-042, this study was excluded from the base case. The PD-L1 IHC 22C3 DAKO pharmDx assay, which is the most commonly used assay in clinical practice,^70^ was used to determine the patient eligibility in KEYNOTE-024, KEYNOTE-042 and its China extension, and EMPOWER-Lung 1.^33,71^ IMpower110 used the Ventana SP142 IHC platform as the primary method for PD-L1 detection to determine the eligibility. This method relied on the staining of both tumor cells and tumor-infiltrating immune cells.^72^ The comparability and interchangeability of the 22C3 DAKO pharmDx and Ventana SP142 assays have been evaluated in the literature, and multiple independent studies have demonstrated strong discordance between these two assays in measuring PD-L1 expression levels. PD-L1 level is highly associated with the degree of response and survival benefit.^24^ The large difference of ORRs observed in patients diagnosed with advanced NSCLC treated with atezolizumab between IMpower110 (38.2% ORR with SP142) and CITYSCAPE (24% ORR with 22C3) may have been the result of different assays being used when selecting patients.^55,73^ Therefore, any comparison between patients receiving cemiplimab in EMPOWER-Lung 1 and atezolizumab in IMpower110 would have included patients with differing PD-L1 levels. Consequently, IMpower110 was also excluded from the base case. The MYSTIC trial (reporting data on durvalumab monotherapy) was excluded because durvalumab was determined not to be a relevant comparator for NSCLC with PD-L1 ⩾50% as described earlier.^56,74^

For survival outcomes (OS and PFS), FE fractional polynomial model NMAs were performed as the base case analysis because three trials violated the proportional hazards assumption. In addition, constant HRs in NMAs were not representative of survival data (time-to-event outcomes) involving immune-oncology trials. Rahman *et al*.^75^ found that a sizable proportion of time-to-event outcomes reported in oncology clinical trials across various solid tumor types showed evidence of deviations from proportional hazards (~25%), concordant with prior estimates. Based on this frequency, reporting of summaries from the Grambsch–Therneau or other tests to quantify the evidence of deviations from proportional hazards and visualizations of HR variations over time (e.g. Schoenfeld residual plots) may be used when presenting trial results. If HR variations over time indicate non-monotonic time-dependent treatment effects (HRs over time), then the evaluation and estimation of treatment effects requires complex statistical procedures, like those in these analyses.^75^ In our study, however, there were no qualitative differences in inference between the models.

An indirect comparison of immunotherapies among patients with locally advanced or metastatic NSCLC with PD-L1 expression ⩾50% who had not received prior systemic therapy for their locally advanced or metastatic disease was recently published.^76^ Majem *et al.* assessed OS and PFS with a constant HR NMA using generalized pairwise modeling framework with the Bucher method, which ignored the nonproportionality observed in the survival data. For OS, results of cemiplimab *versus* pembrolizumab and atezolizumab were generally consistent with the constant HR NMA reported in the Supplemental material. For PFS, Majem *et al.* assessed pembrolizumab separately for KEYNOTE-024 and KEYNOTE-042 (i.e. two separate nodes in network), with the justification that significant heterogeneity (*I*^2^ = 80.7%, *p* = 0.006) was determined between the KEYNOTE trials; however, the authors did not identify obvious clinical heterogeneity to prevent pooling the KEYNOTE trials for the OS analysis. Sensitivity analyses with each KEYNOTE trial individually could be conducted in this case, similar to those performed in the current NMA (see Supplemental material). In their network of evidence, conducting a pairwise meta-analysis of KEYNOTE-024 and KEYNOTE-042 would have been appropriate. In the current NMA, a thorough feasibility assessment was conducted, and it was determined that these two trials could be pooled together, as there were no substantial differences in treatment effect modifiers. PFS NMAs excluding individual pembrolizumab trials here produced similar findings to Majem *et al.* for cemiplimab–pembrolizumab comparisons. Regarding trial inclusion, Majem *et al.* did not take any measures to mitigate potential bias due to different PD-L1 detection methods across trials, simply noting these differences as a limitation. They also included several factual inaccuracies in data reporting. For instance, Majem *et al.* incorrectly noted only KEYNOTE-042 included patients with Stage III NSCLC who were not candidates for surgical resection or definitive chemoradiation, or patients with metastatic NSCLC, while EMPOWER-Lung 1 also enrolled patients with Stage IIIB NSCLC. In addition, OS HRs reported for KEYNOTE-024 in the forest plots did not match the source data.^76^

The main strength of the current analysis was the use of robust statistical models for time-varying HRs. NMAs for survival outcomes based on constant HRs relied on the proportional hazard assumption, which was implausible given that this assumption was shown to be violated in several trials for OS and PFS. As an alternative to the constant HR, which is a univariate treatment effect measure, a multivariate treatment effect measure that describes how the relative treatment effect (e.g. HR) developed over time was used in these analyses. By relaxing this proportional hazard assumption and incorporating additional parameters for the treatment effect, the NMA model more closely fit the observed data. Thorough sensitivity analyses were also performed to investigate the impact of excluding KEYNOTE-024 and KEYNOTE-042, and long-term data reported by KEYNOTE-024. Overall, findings were relatively consistent across the sensitivity and base case analyses, providing evidence for the robustness of study results. All analyses in the base case only included data from the 22C3 DAKO pharmDx assay, minimizing the clinical heterogeneity contributed by discordance between assays. All included trials were published within the past 5 years, representing the current monotherapy treatment landscape for patients with high PD-L1 expression. A limitation of the NMA was the small number of trials per direct comparison in the networks, with each pair of interventions (nodes) informed by only one or two trials. This resulted in relatively little data being available for each comparison; consequently, estimated HRs had greater uncertainty (i.e. wider CrIs). There were no closed loops in any evidence networks, so it was not feasible to assess the consistency between direct and indirect comparisons. Trial designs differed and locations varied in the base case studies. Two of the RCTs in the evidence base allowed for crossover (EMPOWER-Lung 1 and KEYNOTE-024), but the NMA presented here included OS data that were unadjusted for treatment switching. Crossover posed a risk of bias against interventions under investigation as treatment switching could lessen the observed treatment effects between interventions relative to what would have been observed had no switching taken place. EMPOWER-Lung 1 had a higher proportion of patients with Stage III NSCLC than the other studies included in the NMA. Although the OS and PFS subgroup results from EMPOWER-Lung 1 had overlapping 95% CIs to suggest that disease stage was not a treatment effect modifier,^24^ future research with larger populations should further evaluate the potential impact of disease stage on the efficacy of IO monotherapies in patients with high PD-L1 expression.

The current SLR focused only on IO monotherapies that were licensed or in the process of being evaluated by the FDA for patients with high PD-L1 expression. Nivolumab monotherapy was excluded from the SLR since it was not indicated for the target population with high PD-L1 expression, given the unfavorable efficacy results from the CheckMate 026 trial.^77^ Future analyses should consider the inclusion of IO combination regimens to assess whether the addition of chemotherapy is beneficial for patients with high PD-L1 expression as there have been discrepancies in the results from several recently published RCTs^78–81^ and results from indirect comparisons^82–84^ suggest a potential benefit of IO combination regimens for particular subgroups. However, these other analyses also confirmed the efficacy of single-agent IO as a valid option for the treatment of patients with PD-L1 ⩾50% and no known genomic tumor aberrations. Given that the treatment landscape for advanced and metastatic NSCLC is evolving, this NMA should be updated when new data become available. In addition, future NMAs should compare the impact of inhibitors across different PD-L1 expressions given such levels may impact efficacy.^85^

The limitation of indirect treatment comparisons and extrapolations in this NMA should be noted. While best practices were followed to account for between-study differences, there remains uncertainty whether any unknown or unmeasured prognostic factors or treatment effect modifiers were missing from the models that might influence the outcomes of interest. Head-to-head comparisons for cemiplimab *versus* pembrolizumab are currently unavailable; hence, caution should be taken in drawing conclusions about relative clinical activity *versus* serving as required inputs for modeling purposes.

## Conclusion

Considering the limitations of indirect treatment comparisons and extrapolations, for first-line treatment in patients with locally advanced or metastatic NSCLC and PD-L1 ⩾50%, cemiplimab monotherapy demonstrated statistically significant improvements in PFS and ORR, comparable OS, and no evidence of differences in Grade 3–5 all-cause AEs, IMAEs, and all-cause DAEs *versus* pembrolizumab monotherapy. At 2 years, numerically more patients receiving cemiplimab were alive *versus* patients receiving pembrolizumab, and significantly more were alive without progression. When compared with IC chemotherapy, cemiplimab demonstrated statistically significant improvements in OS, PFS, and ORR, with a lower incidence of Grade 3–5 all-cause AEs. Results from the sensitivity analyses of OS and PFS were generally consistent with the base case.

## Supplemental Material

sj-docx-1-tam-10.1177_17588359221105024 – Supplemental material for Network meta-analysis of immune-oncology monotherapy as first-line treatment for advanced non-small-cell lung cancer in patients with PD-L1 expression ⩾50% Click here for additional data file. Supplemental material, sj-docx-1-tam-10.1177_17588359221105024 for Network meta-analysis of immune-oncology monotherapy as first-line treatment for advanced non-small-cell lung cancer in patients with PD-L1 expression ⩾50% by Nick Freemantle, Yingxin Xu, Florence R. Wilson, Patricia Guyot, Chieh-I Chen, Sam Keeping, Gerasimos Konidaris, Keith Chan, Andreas Kuznik, Kokuvi Atsou, Emily Glowienka, Jean-Francois Pouliot, Giuseppe Gullo and Petra Rietschel in Therapeutic Advances in Medical Oncology
