# Corrigendum to “Network meta-analysis of immune-oncology monotherapy as first-line treatment for advanced non-small-cell lung cancer in patients with PD-L1 expression ⩾50%”

**DOI:** 10.1177/17588359221127078

**Published:** 2022-09-13

Freemantle N, Xu Y, Wilson FR *et al*. Network meta-analysis of immune-oncology monotherapy as first-line treatment for advanced non-small-cell lung cancer in patients with PD-L1 expression ⩾50%. *Ther Adv Med Oncol* 2022; 14: 1–23. DOI: 10.1177/17588359221105024

In the above-referenced article, the dates given for when the literature search was conducted were incorrect in both the abstract and the methods.

In the abstract, the sentence “A systematic literature review and network meta-analysis (NMA) was conducted to identify/compare the efficacy/safety of cemiplimab versus pembrolizumab or other immune-oncology monotherapies from randomized-controlled trials (RCTs) published in November 2010–2020.” has been corrected to “A systematic literature review and network meta-analysis (NMA) was conducted to identify/compare the efficacy/safety of cemiplimab versus pembrolizumab or other immune-oncology monotherapies from randomized-controlled trials (RCTs) published from January 2010 to November 2020.”

In the section titled ‘Systematic literature review’, the sentence “Only first-line treatments given as monotherapies that were licensed or those that were in the process of being evaluated by the US FDA at the time of the systematic literature review (SLR) initiation (November 2019) were included.” has been corrected to “Only first-line treatments given as monotherapies that were licensed or those that were in the process of being evaluated by the US FDA at the time of the systematic literature review (SLR) initiation (November 2020) were included.”

These sentences have been corrected in the published version of the article.

