# Supplementary material for: Network meta-analysis of immune-oncology monotherapy as first-line treatment for advanced non-small-cell lung cancer in patients with PD-L1 expression ⩾50%
Source: Ther Adv Med Oncol. 2022 Jun 16;14:17588359221105024. doi: 10.1177/17588359221105024 (PMC9210099; doi:10.1177/17588359221105024)
Supplement: sj-docx-1-tam-10.1177_17588359221105024 – Supplemental material for Network meta-analysis of immune-oncology monotherapy as first-line treatment for advanced non-small-cell lung cancer in patients with PD-L1 expression ⩾50% [file sj-docx-1-tam-10.1177_17588359221105024.docx]

# Supplementary materials

## FDA Indications for cemiplimab, pembrolizumab, and atezolizumab

| **Treatment** | **First-line indication for advanced NSCLC** |
| --- | --- |
| Cemiplimab^90^ | - for the first-line treatment of patients with NSCLC whose tumors have high **PD-L1 expression (TPS ≥50%)** as determined by an FDA-approved test, with no *EGFR*, *ALK*, or *ROS1* aberrations, and is: - **locally advanced** where patients are not candidates for surgical resection or definitive chemoradiation or - **metastatic.** |
| Pembrolizumab^20^ | - in combination with pemetrexed and platinum chemotherapy, as first-line treatment of patients with **metastatic non-squamous NSCLC**, with no *EGFR* or *ALK* genomic tumor aberrations. - in combination with carboplatin and either paclitaxel or paclitaxel protein-bound, as first-line treatment of patients with **metastatic squamous NSCLC.** - as a single agent for the first-line treatment of patients with NSCLC expressing **PD-L1 (TPS ≥1%)** as determined by an FDA-approved test, with no EGFR or ALK genomic tumor aberrations, and is:   - Stage III where patients are not candidates for surgical resection or definitive chemoradiation, or   - metastatic. - as a single agent for the treatment of patients with **metastatic NSCLC** whose tumors express **PD-L1 (TPS ≥1%)** as determined by an FDA-approved test, with disease progression on or after platinum-containing chemotherapy. Patients with EGFR or ALK genomic tumor aberrations should have disease progression on FDA-approved therapy for these aberrations prior to receiving pembrolizumab. |
| Atezolizumab^22^ | - for the first-line treatment of adult patients with **metastatic NSCLC** whose tumors have high PD-L1 expression (PD-L1 stained **≥50% of tumor cells** or PD-L1 stained tumor-infiltrating **immune cells covering ≥10%** of the tumor area), as determined by an FDA-approved test, with no *EGFR* or *ALK* genomic tumor aberrations. - in combination with bevacizumab, paclitaxel, and carboplatin, for the first-line treatment of adult patients with metastatic non-squamous NSCLC with no *EGFR* or *ALK* genomic tumor aberrations. - in combination with paclitaxel protein-bound and carboplatin for the first-line treatment of adult patients with **metastatic non-squamous NSCLC** with no *EGFR* or *ALK* genomic tumor aberrations. - for the treatment of adult patients with **metastatic NSCLC** who have disease progression during or following platinum-containing chemotherapy. Patients with *EGFR* or ALK genomic tumor aberrations should have disease progression on FDA-approved therapy for NSCLC harboring these aberrations prior to receiving atezolizumab. |

**Medline Search**

| **Database: Ovid MEDLINE(R) and Epub Ahead of Print, In-Process & Other Non-Indexed Citations, Daily and Versions(R) 1946 to November 17, 2020**  **Search executed on November 18, 2020** | | | |
| --- | --- | --- | --- |
| **#** | **Criteria** | **Search terms** | **Results** |
| 1 | Population terms | exp Carcinoma, non-small-cell-lung/ | 54,464 |
| 2 |  | ((((non adj2 small adj2 cell) or (bronchial adj3 non adj2 small adj2 cell)) adj3 (cancer or carcinoma)) or NSCLC).ti,ab. | 67,549 |
| 3 |  | or/1-2 | 77,785 |
| 4 |  | ("stage iiib" or "stage iiic" or "stage iii b" or "stage iii c" or "stage iv" or metast* or disseminat* or spread* or migration* or advanced or progress* or invasive or aggressive or unresect* or "not operable" or inoperable or untreatable or "not treatable" or secondary or recurrent or incurable or "not curable").mp. | 3,866,157 |
| 5 |  | 3 and 4 | 45,674 |
| 6 | Intervention terms | (cemiplimab or Libtayo or REGN2810 or REGN-2810 or R2810).mp. | 101 |
| 7 |  | exp immunotherapy/ | 280,115 |
| 8 |  | (PD-1 or PD-L1 or (programmed adj3 death) or immunotherapy or (checkpoint adj2 inhibitor*)).mp. | 148,946 |
| 9 |  | (Pembrolizumab or MK-3475 or MK3475 or lambrolizumab or Keytruda).mp. | 4679 |
| 10 |  | (Atezolizumab or MPDL-3280A or MPDL3280A or Tecentriq).mp. | 1321 |
| 11 |  | (Avelumab or MSB-0010718C or MSB0010718C or Bavencio).mp. | 521 |
| 12 |  | (Durvalumab or MEDI-4736 or MEDI4736 or Imfinzi).mp. | 640 |
| 13 |  | (tislelizumab or BGB-A317 or BGBA317).mp. | 26 |
| 14 |  | (AK105 or AK-105).mp. | 1 |
| 15 |  | or/7-14 | 363,579 |
| 16 |  | exp Cisplatin/ | 52,518 |
| 17 |  | exp Carboplatin/ | 11,754 |
| 18 |  | (cisplatin or platinol or carboplatin or paraplatin).mp. | 88,476 |
| 19 |  | or/16-18 | 88,476 |
| 20 |  | exp taxol/ | 27,074 |
| 21 |  | exp Paclitaxel/ | 27,074 |
| 22 |  | exp Docetaxel/ | 10,722 |
| 23 |  | exp Pemetrexed/ | 2091 |
| 24 |  | exp Vinblastine/ | 12,613 |
| 25 |  | exp vinorelbine/ | 2712 |
| 26 |  | exp Etoposide/ | 16,754 |
| 27 |  | exp Ifosfamide/ | 4806 |
| 28 |  | (paclitaxel or nab-paclitaxel or abraxane or taxane or taxol or onxol or docetaxel or taxotere or pemetrexed or alimta or gemcitabine or gemzar or vinblastine or velbe or velban or vinorelbine or navelbine or NVB or etoposide or etopophos or VP-16 or VP16 or ifosfamide or ifex or IFO).mp. | 116,121 |
| 29 |  | or/20-28 | 116,121 |
| 30 |  | 19 and 29 | 31,416 |
| 31 |  | (gemcitabine or gemzar).mp. | 17,527 |
| 32 |  | exp Docetaxel/ or exp vinorelbine/ | 13,047 |
| 33 |  | (taxane or docetaxel or taxotere or vinblastine or velbe or velban or vinorelbine or navelbine or NVB).mp. | 38,365 |
| 34 |  | or/32-33 | 38,365 |
| 35 |  | 31 and 34 | 2976 |
| 36 |  | 15 or 29 or 30 or 35 | 476,093 |
| 37 | SIGN filters for RCTs | exp randomized controlled trials as topic/ | 141,373 |
| 38 |  | exp randomized controlled trial/ | 518,339 |
| 39 |  | exp random allocation/ | 104,055 |
| 40 |  | exp double blind method/ | 160,904 |
| 41 |  | exp single blind method/ | 29,310 |
| 42 |  | exp clinical trial/ | 875,201 |
| 43 |  | clinical trial, phase i.pt. | 21,001 |
| 44 |  | clinical trial, phase ii.pt. | 33,737 |
| 45 |  | clinical trial, phase iii.pt. | 17,493 |
| 46 |  | clinical trial, phase iv.pt. | 1994 |
| 47 |  | controlled clinical trial.pt. | 93,935 |
| 48 |  | randomized controlled trial.pt. | 517,352 |
| 49 |  | multicenter study.pt. | 282,998 |
| 50 |  | clinical trial.pt. | 525,877 |
| 51 |  | exp clinical trials as topic/ | 348,759 |
| 52 |  | or/37-51 | 1,393,270 |
| 53 |  | (clinical adj trial$).tw. | 380,341 |
| 54 |  | ((singl$ or doubl$ or treb$ or tripl$) adj (blind$3 or mask$3)).tw. | 175,776 |
| 55 |  | exp placebos/ | 35,197 |
| 56 |  | placebo$.tw. | 219,604 |
| 57 |  | randomly allocated.tw. | 29,722 |
| 58 |  | (allocated adj2 random$).tw. | 33,076 |
| 59 |  | or/53-58 | 653,903 |
| 60 |  | 52 or 59 | 1,671,167 |
| 61 |  | exp Case study/ | 2,135,548 |
| 62 |  | Case report.tw. | 320,593 |
| 63 |  | exp report/ or exp letter/ | 1,112,043 |
| 64 |  | or/61-63 | 3,089,610 |
| 65 |  | 60 not 64 | 1,625,200 |
| 66 |  | letter.pt. | 1,108,724 |
| 67 |  | editorial.pt. | 547,425 |
| 68 | Other study design terms | exp retrospective studies/ | 851,848 |
| 69 |  | exp cohort studies/ | 2,055,402 |
| 70 |  | exp cohort analysis/ | 2,055,402 |
| 71 |  | (observational adj3 (study or studies or design or analysis or analyses)).ti,ab. | 153,499 |
| 72 |  | (retrospective adj7 (study or studies or design or analysis or analyses or cohort or data or review)).ti,ab. | 509,073 |
| 73 |  | ((comment or editorial or practice-guideline or review or letter or journal correspondence or posters or News or Newspaper article or meeting abstracts or lectures or interview or historical article or handbooks or guidelines or guidebooks or essays or editorial or database or comment or clinical conference or catalogs) not "randomized controlled trial").pt. | 5,160,731 |
| 74 |  | or/66-73 | 7,307,836 |
| 75 |  | 65 not 74 | 1,012,310 |
| 76 |  | exp Meta Analysis/ | 122,463 |
| 77 |  | (meta adj (analy$ or metaanalys$)).tw. | 184,844 |
| 78 |  | (systematic adj (review$1 or overview$1)).tw. | 185,923 |
| 79 |  | (cochrane or embase or psychlit or psyclit or psychinfo or psycinfo or cinahl or cinhal or science citation index or "bids" or reference lists or bibliograph$ or hand-search$ or manual search$ or relevant journals or data extraction).ab. | 192,105 |
| 80 |  | review.pt. | 2,719,293 |
| 81 |  | or/76-80 | 2,911,600 |
| 82 |  | 75 not 81 | 978,286 |
| 83 |  | exp animal/ | 23,595,717 |
| 84 |  | exp human/ | 18,838,297 |
| 85 |  | 83 not (83 and 84) | 4,757,420 |
| 86 |  | 82 not 85 | 889,026 |
| 87 | Combined criteria | 6 or 36 | 476,109 |
| 88 |  | 5 and 86 and 87 | 3143 |
| 89 | Language restriction | limit 88 to english language | 2971 |
| 90 | Date restriction | limit 89 to yr="1980 -Current" | 2971 |

## Statistical methods

Conventional NMAs for survival outcomes were based on HR estimates and relied on the proportional hazard assumption, which is biased if the hazard functions of competing interventions cross. The proportional hazard assumption regarding time-to-event outcomes for each individual trial was assessed using the Grambsch-Therneau test.^91^ The hazard function describes the instantaneous event (e.g., death) rate at any point in time. Ouwens et al. and Jansen have presented methods for an NMA of survival data using a multidimensional treatment effect as an alternative to the synthesis of constant HRs.^92,93^ The hazard functions of the interventions in a trial are modeled using known parametric survival functions or fractional polynomials. The difference in the parameters was considered the multidimensional treatment effect, which is synthesized (and indirectly compared) across studies. With this approach, the treatment effects are represented by multiple parameters rather than a single parameter. The model introduced by Jansen was used for the NMAs of OS and PFS.^93,94^

For OS and PFS in the base case analysis, the following competing survival distributions were considered using the multivariate NMA framework: Weibull, Gompertz, and second-order fractional polynomials including p1=0 or 1 and p2=–1, −0.5, 0, 0.5, or 1. In essence, these second-order fractional polynomial models were extensions of the Weibull and Gompertz model and allow arc- and bathtub-shaped hazard functions, which emulate parametric distributions such as log-normal and log-logistic. For the relative treatment effects in the second-order fractional polynomial framework, models were assessed which assumed: (1) treatment only has an impact on two of the three parameters describing the hazard function over time (i.e., one scale and one shape parameter), and (2) treatment has an impact on one parameter describing the hazard function over time (i.e., scale only parameter). For time-to-event outcomes assuming time-varying treatment effects, results are presented in terms of HRs until the maximum follow-up of the included trials.

For binary outcomes (e.g., ORR, Grade 3–5 AEs), NMAs were performed based on the proportion of patients experiencing the event of interest using a logistic regression model with a binomial likelihood and logit link.

For the sensitivity analyses, the NMA of reported HRs in terms of OS and PFS (assuming proportional hazards between treatments) was performed using a regression model with a contrast-based normal likelihood for the log HR (and corresponding standard error) of each trial (or comparison) in the network according to the framework outlined by Dias et al.^95^

## Sensitivity analysis including IMpower110

IMpower110 was not included in the base case analysis because the primary IHC assay used to measure PD-L1 expression differed from the other trials. Since OS and PFS results were available for the PD-L1 TPS ≥50% population measured using the 22C3 assay in a prespecified subgroup analysis, such data were considered in the sensitivity analysis. However, all patients in IMpower110 were selected using the SP142 assay, and then retested with 22C3 for PD-L1 ≥50% where about 20% did not have similar levels of PD-L1 given the differences between the assays. Patients initially tested with the SP142 assay likely had a high PD-L1 expression of ~80–98% when tested with the 22C3 assay used in EMPOWER Lung-1.^17,23,61,85^ Therefore, caution is required when interpreting the results of the sensitivity analysis, as PD-L1 level is highly associated with response and is a known treatment effect modifier of immune-oncology therapies.

NMAs were performed for OS using fractional polynomial models and constant HRs. PFS Kaplan-Meier curves for IMpower110 were not available for the PD-L1 TPS ≥50% population based on the 22C3 assay; thus, the fractional polynomial NMA for PFS was not feasible. The ORR was only reported for the population with tumor cells ≥50% or immune cells ≥10% PD-L1–positive; therefore, the ORR analysis was not performed. The cemiplimab population used for this subanalysis was the PD-L1 ≥50% population.

Results for the comparisons of cemiplimab versus IC chemotherapy and pembrolizumab were very similar to the results from the corresponding analyses without IMpower110. For OS, according to the model selection process, the best-fitting model was the same as the base case analysis, an FE second-order fractional polynomial with p1=1 and p2=–0.5 (scale and second shape). Cemiplimab was consistently associated with a statistically significant improvement in OS compared with IC chemotherapy with the OS benefit increasing steadily over time (average time-varying HR [95% CrI]: 0.55 [0.40–0.75], 1–12 months; 0.40 [0.25–0.62], 13–24 months; 0.37 [0.22–0.61], 25–30 months; 0.39 [0.24–0.62], 13–30 months). Cemiplimab was associated with an improvement in OS compared with atezolizumab, although results were not statistically significant (average time-varying HR [95% CrI]: 0.87 [0.54–1.41], 1–12 months; 0.84 [0.42–1.69], 13–24 months; 0.84 [0.39–1.80], 25–30 months; 0.84 [0.41–1.73], 13–30 months)

## Sensitivity analyses including longest follow-up data from base case trials

This analysis explored the inclusion of the longest follow-up data from trials. At the time of the SLR update (searches executed in November 2020), results were available from the 5-year follow-up analysis of KEYNOTE-024 (median follow-up of 59.9 months) in the form of conference slides, which represented the most mature data from this trial. Data available from EMPOWER-Lung 1 and KEYNOTE-042 were the same as those included in the base case analysis. Analyses were performed for OS and PFS using fractional polynomial models and constant HRs and were based on the PD-L1 ≥50% population from EMPOWER-Lung 1. Analyses were performed for the network including three trials (EMPOWER-Lung 1, KEYNOTE-024, and KEYNOTE-042) in alignment with the base case analyses, and for the network excluding KEYNOTE-042, given that extended follow-up data for this trial were not available at the time of the SLR update.

An FE fractional polynomial model NMA was performed to assess OS for cemiplimab versus competing interventions based on the base case evidence network including three trials. According to the model selection process, the best-fitting second-order time-varying HR model was the FE second-order fractional polynomial with p1=1 and p2=–0.5 (scale and second shape). Results of time-varying OS HRs and HRs by 12-month time period for cemiplimab versus competing interventions are presented in Table S1. Results showed an improvement in OS with cemiplimab compared with IC chemotherapy and pembrolizumab. Cemiplimab was consistently associated with a statistically significant improvement in OS compared with IC chemotherapy with the OS benefit increasing steadily over time from month 3 (HR 0.64, 95% CrI 0.46–0.89) through month 30 (HR 0.37, 95% CrI 0.22–0.61). Cemiplimab was also associated with an improvement in OS compared with pembrolizumab (3 months: HR 0.80, 95% CrI 0.54–1.19; 30 months: HR 0.68, 95% CrI 0.39–1.18), although results were not statistically significant. These results were consistent with the base case analysis, which used data from the final analysis of KEYNOTE-024, and results were also consistent with the FE NMA based on constant HRs.

## Table S1. Estimated OS HRs over time for cemiplimab versus competing interventions from scenario with longest follow-up data for base case network including three trials

| **Cemiplimab  vs** | **Time-varying HR (95% CrI)** | | | | | | |
| --- | --- | --- | --- | --- | --- | --- | --- |
|  | **3 months** | **6 months** | **9 months** | **12 months** | **18 months** | **24 months** | **30 months** |
| **IC chemotherapy** | **0.64 (0.46–0.89)** | **0.51 (0.36–0.70)** | **0.46 (0.31–0.66)** | **0.43 (0.29–0.64)** | **0.40 (0.25–0.63)** | **0.38 (0.23–0.62)** | **0.37 (0.22–0.61)** |
| **Pembrolizumab** | 0.80 (0.54–1.19) | 0.75 (0.52–1.07) | 0.72 (0.48–1.08) | 0.71 (0.45–1.10) | 0.69 (0.42–1.14) | 0.68 (0.40–1.16) | 0.68 (0.39–1.18) |

| **Cemiplimab**  **vs** | **Average time-varying HR (95% CrI) within time period** | | | |
| --- | --- | --- | --- | --- |
|  | **1–12 months** | **13–24 months** | **25–30 months** | **13–30 months** |
| **IC chemotherapy** | **0.55 (0.40–0.75)** | **0.40 (0.25–0.63)** | **0.37 (0.23–0.62)** | **0.39 (0.24–0.62)** |
| **Pembrolizumab** | 0.76 (0.53–1.09) | 0.69 (0.42–1.14) | 0.68 (0.39–1.18) | 0.69 (0.41–1.15) |

From FE fractional polynomial NMA (p1=1, p2=–0.5, scale and second shape). All bolded values are statistically significant at the 0.05 significance level.
CrI, credible interval; FE, fixed effect; HR, hazard ratio; IC, investigator’s choice; NMA, network meta-analysis; OS, overall survival.
